# Reading for pleasure in childhood and adolescent healthy behaviours: Longitudinal associations using the Millennium Cohort Study

**DOI:** 10.1016/j.ypmed.2019.105889

**Published:** 2020-01

**Authors:** Hei Wan Mak, Daisy Fancourt

**Affiliations:** Department of Behavioural Science and Health, University College London, UK

**Keywords:** Reading for pleasure, Healthy behaviours, Longitudinal study, Children, Development

## Abstract

Leading a heathy lifestyle in adolescence is vital to individual health in later life. Drawing upon various existing theories, this study hypothesised that engagement in reading for pleasure may enhance healthy behaviours amongst young people. Data were analysed from 11,180 children in the UK Millennium Cohort Study and logistic regressions were used to examine the association between reading frequency at age 11 and health behaviours at age 14. Reading most days was associated with lower odds of trying a cigarette and alcohol and a higher likelihood of having two portions of fruit per day independent of confounding factors. However, spending more time reading was associated with less time spent engaging in moderate to vigorous physical activity. Our findings suggest the importance of further studies exploring the potential health benefits of reading amongst young people.

## Highlights

• Reading aged 11 is associated with increased odds of fruit consumption aged 14.
• Reading aged 11 is associated with lower odds of cigarette & alcohol use initiation.
• Reading aged 11 is associated with reduced odds of physical activity engagement.
• Demography, child development and peer influences partly explain associations.

## 1 Introduction

The establishment of a healthy lifestyle has its origin in early life and is a key contributor to health in later life (Ford et al., 2008). In relation to *health-impairing* behaviours, research has provided evidence for the positive relationship between early substance use initiation and smoking, drinking, and drug using and later substance use problems (Dawson et al., 2008; DeWit et al., 2000; Hingson et al., 2006; Merline et al., 2004). In relation to *health-promoting* behaviours, there has been increasing interest in fruit and vegetable consumption and physical activity in childhood given rising levels of childhood obesity (NHS Digital, 2018a; Serdula et al., 1993; Townsend et al., 2013). As a result, there is a need to identify activities that help to promote healthy lifestyles in childhood and adolescence. This study explores the potential role of reading for pleasure in supporting positive and healthy child development.

Many studies have identified factors that predict both health-impairing and healthy behaviours in young people. Borrowing models from Stone et al. (2012) and Degenhardt et al. (2016) on the determinants of substance use, three domains can explain the health behaviours amongst young people: fixed markers of risk (e.g. gender, ethnicity, socio-economic status (SES)), contextual risk factors (e.g. aspects of the social and cultural environment), and individual characteristics and behaviours (e.g. young people's academic achievement, parent-child relationship, and children's mental and psychological well-being). While fixed markers and contextual risk factors are factors that can be challenging to change, *individual characteristics and behaviours* may be modifiable. For instance, it has been shown that adolescents with high school performance consume more fruit and vegetable than those with lower school performance (Doku et al., 2011). Parent-child relationship, family cohesion, and parent-child communication are also associated with children's early substance use initiation (Garcia and Gracia, 2009; Velleman et al., 2005), and their physical activity and healthy diet (Niu et al., 2018; Ornelas et al., 2007). Other studies have shown correlations between childhood mental and psychological well-being and alcohol problems (Marmorstein, 2009) and cannabis use (von Sydow et al., 2002). However, for most children, these factors can still be hard to control. So there is a recognised need to identify further factors that could encourage healthy behaviours in children.

One potential behaviour that enables young people to change their behaviour themselves is engagement in reading for pleasure, which allows them to gain knowledge and to be open to change by entering into different imaginary worlds (Djikic et al., 2012). There are a number of theories suggesting that reading for pleasure may *directly* exert beneficial effects on young people's healthy behaviours. For example, theory of mind (the ability to understand others' feelings and emotions (Shamay-Tsoory and Aharon-Peretz, 2007)) posits that reading increases individuals' empathy (Bal and Veltkamp, 2013; Mar and Oatley, 2008; Mar et al., 2009; Scales et al., 2000), which can foster prosocial behaviours and inhibit antisocial acts (Lonigro et al., 2014). Further, learning and cognitive theories suggest that individuals learn and change their behaviours according to the models they can identify with from the reading materials (Rousset et al., 2008). One consequence of this can be the building of self-efficacy through showing the narrative development of characters to whom readers can relate. Self-efficacy is a key to the adoption and maintenance of of healthier and less risky behaviours (Kwasnicka et al., 2016). Another consequence is that reading fiction in which prosocial behaviour is normalised whereas risk-taking behaviours are discouraged could directly influence children's behaviours. In addition, it has been shown that if children's knowledge about nutrition and health is improved (e.g. through reading books), this is a significant predictor of fruit and vegetables intake for children in ages 13–15 (Zabinski et al., 2006). *Indirectly*, reading could also encourage healthy behaviours in young people by enhancing academic success (OECD, 2002) and reducing the risk of depression (Dowrick et al., 2012), which are in turn associated with a healthy diet (Doku et al., 2011; Gillen et al., 2012), lower cigarette, alcohol, and drug use (Stone et al., 2012), and higher physical activity (Roshanaei-moghaddam et al., 2009).

Therefore, this study extends the current literature by using a nationally representative data set – the Millennium Cohort Study (MCS) - to explore whether there is a longitudinal association between reading frequency at age 11 (when self-identity starts to develop and becomes more important in behavioural choices (Kronger, 2007)) and health-related behaviours at age 14; the age when children typically initiate substance use (NHS Digital, 2018b; NHS Digital, 2018c) and reduce their levels of fruit consumption and physical activity (Townsend et al., 2013; Cooke and Wardle, 2005; Nader et al., 2008; Trost et al., 2002). Given that a broad range of factors could confound such an association, this analysis also explored whether any associations could be explained by factors including baseline health behaviours, demographic and socio-economic factors, aspects of children's personal and academic development, child mental health and wellbeing, parent-child relationship and patterns of engagement, and parental and peer influences on reading and health behaviours.

## 2 Methods

### 2.1 Participants

MCS is a nationally-representative, longitudinal study that follows the lives of 19,000 individuals who were born in the UK in 2000–2001. The cohort has been followed at ages 3, 5, 7, 11, 14, and 17 (the 17-year follow-up was not available at the time of writing), with interviews with cohort members, parents, and teachers assessing cohort members' physical and psychological development, academic performance, daily life experiences, and demographic backgrounds. We used data from Sweep 5 (2011; response 81%) and Sweep 6 (2015; response 76%) interviews when the cohort was aged 11 and 14 respectively.

A total of 13,469 children were included in Sweep 5. In our analysis, we only included participants who were not born as twins or triplets (to minimise the complexity of considering differences between fraternal or dizygotic twins; <1.5% of the whole sample were born as twins or triplets) (*n* = 13,287), who lived with natural parents (*n* = 12,889), and who answered all the outcome variables measured at Sweep 6 (*n* = 11,180). A flowchart of the analytical sample is shown in Supplementary Fig. 1. The MCS receives ethical approval from the National Research Ethics Service and all participants provide informed consent.

### 2.2 Measures

Children's reading for pleasure is differentiated in the data set from reading for educational purposes and was measured in Sweep 5 through self-report by children themselves. The indicator was a 5-point scale, ranging from “never”, “less often than once a month”, “at least once a month”, “at least once a week”, to “most days”.

We explored four health behaviours measured in Sweep 6: smoking, drinking alcohol, fruit consumption, and physical activity. For smoking and drinking alcohol, two binary variables were generated for whether the cohort members themselves had ever used these substances by age 14. Fruit consumption was measured by asking children how often the children had at least two portions of fruit per day (e.g. a whole piece of fruit, a fruit salad; fruit juices were not considered). Two portions of fruit every day is recommended as an adequate level by the US Department of Agriculture (2019). Physical activity was measured by asking children the number of days in the past week they spent doing at least an hour of moderate to vigorous physical activity, such as riding a bike and running. We defined an adequate level of physical activity as doing some physical activity five or more days per week; the recommended amount of moderate to vigorous physical activity for children suggested by UK Expert Consensus Group (Pate et al., 2002). For fruit consumption and physical activity, data were also available at Sweep 5 (answered by parents on behalf of their children for fruit consumption and children themselves for physical activity) so we controlled for these baseline healthy behaviours in the relevant models.

To take advantage of the rich data set, as well as to ensure the robustness of the results, we controlled for all identified confounding variables in Sweep 5. Demographic factors included children's gender and ethnicity (white vs other); parents' education (NVQ level 1 vs level 2 vs level 3 vs level 4 vs level 5 vs Overseas/none of these); household income; parents' employment status (semi-routine/routine vs lower supervisory/lower technician vs small employers/self-employed vs intermediate vs managerial/professional) and parents' marital status (married/remarried/in a civil partnership vs single, never married and never in a civil partnership vs legally separated/divorced/widowed/in a surviving civil partnership).

Child development factors included children's behaviours (prosocial behaviour, emotional problems, peer problems, conduct problems, and hyperactivity/inattention, measured using the 25-item Strengths and Difficulties Questionnaire; all standardised) (Goodman, 1997), and children's educational performance (using an index constructed from teachers' rating on Maths, English, and Science, summed and standardised).

Children's mental health included depressive symptoms (an index of five items assessing depressive symptoms, *α*=0.65; standardised); subjective well-being (a six-item measure of children reported being happy with their life as a whole, school, school work, family, friends, and appearance; *α*=0.83; standardised) (ONS, 2013), and self-esteem (using Rosenberg's five-item self-esteem scale, *α*=0.83; standardised) (Rosenberg, 1979).

Familial factors included parents' self-rated closeness of relationship and frequency of arguments with children (all standardised) and frequency of playing active games with parents.

Peer influence included the number of books parents had in the home (a six-point scale ranged from “0–10”, “11–25”, “26–100”, “101–200”, “201–500”, “more than 500”), the frequency of parents' reading for pleasure (a seven-point scale ranged from “less often or never”, “at least once a year”, “every few months”, “at least once a month”, “once or twice a week”, “several times a week”, “every day or almost every day”), the frequency of children going to the library (a seven-point scale) and the frequency of visiting a social networking website (e.g. Facebook) (a five-point scale ranged from “never”, “less often than once a month”, “at least once a month”, “at least once a week”, “most days”). In our models for cigarette use we additionally included parental cigarette use (yes vs no) and peer cigarette use (yes vs no). In our models for alcohol use, we additionally included parental alcohol use (2–3 times a week or more vs <2–3 times a week/never drink), and peer alcohol use (yes vs no).

### 2.3 Statistics

15% of the data (the exposure, confounding, outcome variables) were missing, so we used multiple imputation by chained equations to create 50 imputed datasets, using the exposure and confounding variables in the statistical models as predictor variables. Sensitivity analyses using just available data produced comparable results, so we present the imputed datasets for greater statistical power. Baseline differences in demographic factors by frequency of reading were assessed using Chi-square tests. All covariates exhibited low to moderate correlations with each other (i.e. <0.5).

To understand the association between reading for pleasure and children's healthy lifestyles, we used logistic regression models. In order to identify the proportion of the association explained by different factors, we built our models sequentially, calculating the percentage of protective association explained (PPAE) by the inclusion of different factors at each stage (Lin et al., 1997). PPAE = (OR (E + C + X) − OR (E + C))/(1 − OR (E + C)) ∗ 100, where OR = odds ratio, E = exposure, C = covariates, and X = explanatory variable being tested. As a supplementary analysis, we repeated our analyses stratified by gender. All regression assumptions were met and all analyses were conducted in Stata v15.0. All models were weighted using inverse probability weighing to account for differential non-response and to ensure the sample remained representative.

Finally, in this study we explored longitudinal associations while controlling for identified confounders. Some of these factors are time-invariant or not likely to be affected by a child's reading (e.g. parental education level and household income). Others might not just explain the association due to baseline differences but could act as causal mediators (e.g. depression and educational performance). For this reason, we also ran a model that only included factors unlikely to lie on the causal pathway to compare results.

## 3 Results

### 3.1 Demographics

Compared to children with a lower frequency of reading, those who read most days were likely to be female and have parents with a higher level of educational background. Additionally, children who read daily at age 11 showed better psychological and behavioural adjustment and mental health (Table 1).

**Table 1:** Descriptive statistics of the whole sample [weighted].

	Variables	Never read (*N* = 668)	Less often than once a month (*N* = 757)	At least once a month (*N* = 1084)	At least once a week (*N* = 2988)	Most days (*N* = 4883)	*p*-Value
	N(%)/mean (SE)						
Health behaviours, Sweep 6	Ever smoked (yes/no)	147 (22.0%)	129 (17.1%)	159 (14.7%)	472 (15.8%)	576 (11.8%)	0.000
	Ever used alcohol (yes/no)	367 (55.0%)	377 (49.8%)	590 (54.4%)	1554 (52.0%)	2285 (46.8%)	0.000
	Two portions or more of fruit consumption per day (yes/no)	162 (24.3%)	179 (23.7%)	263 (24.3%)	932 (31.2%)	1939 (39.7%)	0.000
	Engaged in at least 60-min moderate to vigorous physical activity 5+ days per day	277 (41.4%)	328 (43.3%)	402 (37.1%)	1222 (40.9%)	1782 (36.5%)	0.001
Demographic factors	Female	206 (30.9%)	266 (35.2%)	455 (42.0%)	1449 (48.5%)	2852 (58.4%)	0.000
	White	606 (90.7%)	683 (90.2%)	988 (91.1%)	2644 (88.5%)	4278 (87.6%)	0.000
	Parent's education						0.001
	NVQ level 1	78 (11.7%)	86 (11.4%)	130 (12.0%)	305 (10.2%)	355 (7.27%)	
	NVQ level 2	244 (36.5%)	251 (33.2%)	346 (31.9%)	965 (32.3%)	1284 (26.3%)	
	NVQ level 3	45 (6.75%)	72 (9.56%)	88 (8.11%)	237 (7.92%)	404 (8.28%)	
	NVQ level 4	114 (17.0%)	178 (23.5%)	266 (24.5%)	786 (26.3%)	1587 (32.5%)	
	NVQ level 5	30 (4.47%)	36 (4.72%)	86 (7.91%)	256 (8.56%)	679 (13.9%)	
	Overseas/none of these	158 (23.6%)	134 (17.7%)	168 (15.5%)	439 (14.7%)	566 (11.6%)	
	Parents' employment status						0.000
	Semi-routine/routine	195 (29.2%)	191 (25.2%)	275 (25.4%)	729 (24.4%)	933 (19.1%)	
	Lower supervisory/lower technician	600 (8.98%)	71 (9.33%)	83 (7.62%)	245 (8.20%)	292 (5.97%)	
	Small employers/self-employed	125 (18.7%)	122 (16.1%)	156 (14.4%)	478 (16.0%)	718 (14.7%)	
	Intermediate	66 (9.88%)	79 (10.5%)	130 (12.0%)	338 (11.3%)	508 (10.4%)	
	Managerial/professional	222 (33.2%)	294 (38.8%)	439 (40.5%)	1198 (40.1%)	2432 (49.8%)	
	The lowest household income bracket (less than £150 per week)	98 (14.7%)	89 (11.7%)	103 (9.95%)	293 (9.82%)	422 (8.64%)	0.000
	Parents' marital status						0.000
	Married/remarried/in a civil partnership	382 (57.2%)	511 (67.5%)	724 (66.8%)	2041 (68.3%)	3589 (73.5%)	
	Single, never married and never in a civil partnership	132 (19.8%)	120 (15.8%)	165 (15.2%)	454 (15.2%)	625 (12.8%)	
	Legally separated/divorced/widowed/in a surviving civil partnership	154 (23.0%)	126 (16.7%)	195 (18.0%)	496 (16.6%)	669 (13.7%)	
Baseline healthy behaviours, Sweep 5	Two portions or more of fruit consumption per day (yes/no)	379 (56.8%)	496 (65.5%)	731 (67.4%)	2142 (71.7%)	3755 (76.9%)	0.000
	Frequency of engaging in moderate to vigorous physical activity most days (yes/no)	391 (58.6%)	397 (52.5%)	588 (54.2%)	1975 (66.1%)	2627 (53.8%)	0.509
Child development factors	Prosocial behaviour (ranges from −5.78 to 0.91)	−0.18 (0.05)	−0.03 (0.04)	0.02 (0.04)	0.04 (0.02)	0.08 (0.02)	0.000
	Emotional problems (ranges from −1.00 to 4.29)	0.12 (0.05)	0.03 (0.04)	−0.02 (0.04)	−0.03 (0.02)	−0.09 (0.02)	0.000
	Peer problems (ranges from −0.93 to 5.42)	0.13 (0.05)	0.04 (0.05)	−0.04 (0.04)	−0.13 (0.02)	−0.07 (0.02)	0.000
	Conduct problems (ranges from −0.87 to 7.28)	0.20 (0.05)	0.07 (0.05)	0.01 (0.04)	−0.06 (0.02)	−0.15 (0.02)	0.000
	Hyperactivity/inattention (ranges from −1.40 to 3.49)	0.36 (0.05)	0.16 (0.04)	0.04 (0.04)	−0.04 (0.02)	−0.21 (0.02)	0.000
	Academic performance (ranges from −2.64 to 1.81)	−0.39 (0.05)	−0.15 (0.04)	−0.02 (0.04)	0.02 (0.02)	0.30 (0.02)	0.000
Children's mental health factors	Depression index (ranges from −1.54 to 5.02)	0.14 (0.05)	0.14 (0.04)	0.03 (0.03)	−0.01 (0.02)	0.05 (0.02)	0.004
	Subjective well-being (ranges from −4.65 to 1.42)	−0.35 (0.05)	−0.23 (0.04)	−0.14 (0.04)	−0.03 (0.02)	0.08 (0.02)	0.000
	Self-esteem (ranges from −5.47 to 1.65)	−0.24 (0.05)	−0.17 (0.04)	−0.14 (0.04)	−0.04 (0.02)	0.07 (0.02)	0.000
Family factors	Closeness with mother (ranges from −4.06 to 0.83)	−0.04 (0.05)	−0.01 (0.04)	−0.04 (0.04)	0.04 (0.02)	−0.01 (0.02)	0.493
	Closeness with father (ranges from −3.19 to 0.94)	0.06 (0.06)	0.00 (0.05)	0.02 (0.04)	0.03 (0.03)	0.00 (0.02)	0.104
	Frequent battles with mother	222 (33.3%)	237 (31.3%)	325 (30.0%)	840 (28.1%)	1318 (27.0%)	0.000
	Frequent battles with father	200 (29.9%)	238 (31.4%)	295 (27.2%)	777 (26.0%)	1274 (26.1%)	0.000
	Play active games with mother at least once or twice a week	145 (21.7%)	192 (25.4%)	304 (28.0%)	908 (30.4%)	1538 (31.5%)	0.000
	Play active games with father at least once or twice a week	277 (41.5%)	369 (48.8%)	491 (45.3%)	1524 (51.0%)	2456 (50.3%)	0.001
Peer influence	Having >200 books at home	76 (11.4%)	120 (15.8%)	162 (14.9%)	538 (18.0%)	1514 (31.0%)	0.000
	High frequency of parents' reading for pleasure (i.e. every day or almost every day)	261 (39.1%)	310 (41.0%)	454 (41.9%)	1360 (45.5%)	2598 (53.2%)	0.000
	Children going to the library at least once a month	90 (13.5%)	137 (18.1%)	207 (19.1%)	669 (22.4%)	1567 (32.1%)	0.000
	Frequency of visiting a social networking website at least once a week	265 (39.7%)	248 (32.7%)	390 (36.0%)	977 (32.7%)	1167 (23.9%)	0.000
	Parents' cigarette use	385 (57.7%)	385 (50.9%)	527 (48.6%)	1300 (43.5%)	1733 (35.5%)	0.000
	Parents' alcohol use (2–3 times or more a week or more)	437 (65.4%)	495 (65.4%)	721 (66.5%)	2026 (67.8%)	3272 (67.0%)	0.614
	Peers' cigarette use	73 (10.9%)	59 (7.85%)	56 (5.12%)	161 (5.38%)	162 (3.32%)	0.000
	Peers' alcohol use	105 (15.7%)	104 (13.8%)	101 (9.29%)	298 (9.97%)	345 (7.06%)	0.000

Notes: The estimated Ns provided are weighted and are the product of the imputated proportions and the number of observations (e.g. the weighted N provided females are 0.309*668 = 206).

### 3.2 Cigarette use

Basic models adjusted only for gender and ethnicity showed that daily reading for pleasure at age 11 was associated with a 16% lower odds of trying a cigarette by the age of 14 (OR = 0.84, 95%CI = 0.80–0.88) (Table 2). This association was in part due to demographic differences in children who did and did not read daily, accounting for 31.3% of the association. It was also partly explained by baseline differences in child development factors (i.e. their psychological and behavioural adjustments and academic performance; 31.3%) and peer influence (e.g. the amount of books the family has at home; 56.3%). Additionally, more modest explanations of the association were due to child mental health (12.5%) and family relationships factors (12.5%). After considering all important factors, the association between reading for pleasure and later cigarette onset was attenuated (OR = 0.97, 95%CI = 0.91–1.02).

**Table 2:** The association between reading at age 11 and health behaviours at age 14, with potential explanatory factors (N = 11,180).

Explanatory factors	Odds ratio	95% CI	PPAE
Cigarette use			
Basic model (gender, ethnicity)	0.84	0.80–0.88	
+ demographic factors	0.89	0.84–0.94	31.3%
+ child development	0.89	0.85–0.94	31.3%
+ child mental health	0.86	0.82–0.91	12.5%
+ family relationships	0.86	0.81–0.90	12.5%
+ peer influence	0.93	0.88–0.98	56.3%
= all	0.97	0.91–1.02	81.3%
Alcohol consumption			
Basic model (gender, ethnicity)	0.94	0.90–0.98	
+ demographic factors	0.95	0.91–0.99	16.0%
+ child development	0.95	0.91–0.98	16.0%
+ child mental health	0.95	0.92–0.99	16.0%
+ family relationships	0.95	0.91–0.98	16.0%
+ peer influence	0.98	0.94–1.02	66.7%
= all	1.00	0.96–1.04	100.0%
Fruit consumption (2+ portions daily)			
Basic model (gender, ethnicity, baseline fruit consumption at age 11)	1.22	1.16–1.28	
+ demographic factors	1.16	1.10–1.22	27.3%
+ child development	1.16	1.11–1.22	27.3%
+ child mental health	1.20	1.14–1.26	9.1%
+ family relationships	1.21	1.15–1.27	4.5%
+ peer influence	1.16	1.10–1.21	27.3%
= all	1.10	1.04–1.15	54.5%
Moderate to vigorous physical activity (5+ days per week)			
Basic model (gender, race, baseline physical activity at age 11)	0.97	0.93–1.01	
+ demographic factors	0.95	0.91–0.99	−66.7%
+ child development	0.97	0.93–1.01	0.00%
+ child mental health	0.96	0.92–1.00	−33.3%
+ family relationships	0.96	0.92–1.01	−33.3%
+ peer influence	0.97	0.93–1.01	0.00%
= all	0.95	0.91–0.99	−66.7%

Note: PPAE: Percentage of Protective Association Explained. Basic model controlled for gender and ethnicity. The model for fruit consumption also controlled for baseline fruit consumption at age 11. The model for physical activity is adjusted for baseline physical activity at age 11. The Demographic Factors model additionally adjusted for parents' education, household income, parents' employment status and parents' marital status. The Child Development model additionally controlled for children's behaviours (prosocial behaviour, emotional problems, peer problems, conduct problems, and hyperactivity/inattention) and children's educational performance. The Child Mental Health model additionally controlled for children's depressive symptoms, subjective well-being and self-esteem. The Family Relationships model additionally controlled for the closeness of parent-child relationship, frequency of arguments between parents and children and frequency of playing active games with parents. The Peer Influence model additionally adjusted for the number of books at home, frequency of parents' reading for pleasure, frequency of children going to the library and frequency of visiting a social networking website. The model for cigarette use is additionally adjusted at the final stage for parental cigarette use and peer cigarette use. The model for alcohol consumption is additionally adjusted at the final stage for parental alcohol use and peer alcohol use.

### 3.3 Alcohol

In our basic models that adjusted only for gender and ethnicity, daily reading for pleasure at age 11 was associated with a 6% lower odds of initiating alcohol use by the age of 14 (OR = 0.94, 95%CI = 0.90–0.98). This association was partly explained by the differences in demographic backgrounds (16.0%), children's development (16.0%), children's mental health (16.0%), family relationships (16.0%) and peer influence (66.7%) amongst those who did and did not read for pleasure daily. These differences accounted for a total of 100% of the association.

### 3.4 Fruit consumption

Reading for pleasure at age 11 was positively and significantly related to children's later fruit consumption. In the basic models where baseline fruit consumption at age 11 was controlled for, reading was associated with a 22% higher odds of having two portions of fruit per day (OR = 1.22, 95%CI = 1.16–1.28). 54.5% of the association was explained by all identified covariates. Amongst all the explanatory variables, demographic factors (such as parents' education and employment status), child development and peer influence accounted for the largest proportion of the association (27.3% each). Nonetheless, the relationship between reading and later fruit consumption remained even after controlling for all identified factors (OR = 1.10, 95%CI = 1.04–1.15).

### 3.5 Physical activity

In contrast to other health behaviours, daily reading for pleasure at age 11 was associated with a 3% decrease in the odds of engaging in moderate to vigorous physical activity at age 14 (albeit not statistically significant). The association between reading and physical activity could not be explained by the covariates, as shown by the smaller size of odds ratios after adjusting for additional variables. The longitudinal relationship between reading and physical activity remained significant after controlling for all the covariates (OR = 0.95, 95%CI = 0.91–0.99).

### 3.6 Sensitivity analyses

Our sensitivity analyses tested potential gender differences (Supplementary Table 1). We generated an interaction term between gender and the frequency of reading for pleasure and included it in the model in addition to all other covariates. We found that the interaction term was only significant in the model estimating fruit consumption (basic model: OR = 1.13, 95%CI = 1.02–1.25; full model: OR = 1.12, 95%CI = 1.01–1.23) and the full model estimating physical activity (OR = 0.94, 95%CI = 0.89–0.99), suggesting that there might be gender differences in the association between reading for pleasure and later fruit consumption and physical activity. To explore this, we reran the analyses on subgroups of females and males and found that the association was stronger for females (full model: OR = 1.15, 95%CI = 1.06–1.25) but still present amongst males (full model: OR = 1.07, 95%CI = 1.00–1.14) in the fruit consumption model. (Supplementary Table 2a). The association between reading for pleasure and physical activity was only significant for males (full model: OR = 0.93, 95%CI = 0.88–0.99) (Supplementary Table 2b).

Finally, when only adjusting for factors that could explain baseline differences but are unlikely to lie on the causal pathway, the relationship was still present for fruit consumption (OR = 1.15, 95%CI = 1.09–1.20) and physical activity (OR = 0.95, 95%CI = 0.91–0.99) as before. Further, there was evidence that there was still an independent relationship for cigarette use (OR = 0.92, 95%CI = 0.87–0.97) (Supplementary Table 3).

## 4 Discussions

In this study, we showed for the first time that reading for pleasure in childhood is associated with healthy behaviours in adolescence. Specifically, we found that reading most days at age 11 is associated with a decreased odds of cigarette and alcohol use, and an increased level of fruit consumption amongst adolescents at age 14, but lower levels of physical activity. For our health-impairing behaviours (cigarette use and alcohol use), the beneficial association was attenuated when considering all factors (such as child mental health and family relationships) and factors relating to peer influence respectively. However, the association for healthy behaviours (fruit consumption and physical activity) was maintained independent of all identified confounders.

Our analyses of potential explanatory factors for these associations were aligned with previous research on predictors of both reading and healthy behaviours. We found that children's demographic background (such as parents' education and household income) explained up to 31.3% of the associations found, which supports previous research suggesting that high SES parents may be more likely to encourage their children both in reading (e.g. access to reading materials, opportunity of parent-child reading) and in establishing a healthy lifestyle (Christensen, 2011; Greaney, 1986; Ross and Mirowsky, 2011), due to factors including available monetary and material resources (Christensen, 2011; Bourdieu, 1984). Similarly, our results also show that children's development, which contains components of children's psychological and behavioural adjustments and academic performance, explain substantial portions of the association between reading and healthy behaviours across models (except the physical activity model). Along with the development of empathy as identified in the previous studies (Djikic et al., 2013; Johnson, 2012), it is possible that reading may also stimulate other positive emotions that support the development of a positive sense of self (Oatley, 2012). In line with previous studies which found a link between reading and academic success (OECD, 2002) and a link between school performance and healthy lifestyle (Doku et al., 2011), children with better school performance may be more likely to be informed on health-related matters, such as the consquences of using cigarettes and the benefits of maintaining a well-balanced diet. It is also plausible that high performing children may tend to read more books, increasing the chance of them being exposed to the reading materials that offer information about healthy eating and behaviours.

Moreover, peer influence (such as parental and peer substance use and reading friendly environment) appeared to explain a large proportion of the relationship between reading and cigarette use and alcohol consumption (explaining the largest amount of the association for these outcomes and also explain more than a quarter of the association for fruit consumption). This suggests both the importance of positive peer influence in encouraging both reading for pleasure and health behaviours (Greaney, 1986), but may also suggest the importance of negative peer influence in reducing engagement with reading and encouraging risk-taking behaviours. The relative influence of both of these types of peer influence on child reading remain to be explored further. Relatedly, family relationships explained some of the association for all outcomes except physical activity, but without such a large association as for peer influence. This result was in line with previous studies that found a positive parent-child relationship and joint family activities were related to a lower probability of children's engagement in risky behaviour (Garmiene et al., 2006; Mak and Iacovou, 2019; Milkie et al., 2015). This suggests that a good family relationship may be pivotal to children's better outcomes (including better mental functioning and healthy activity engagements), particularly during the transition from childhood to adolescence.

However, it is notable that the associations between reading for pleasure and fruit consumption and physical activity were maintained independent of all of these potential explanatory variables. In relation to fruit consumption, as previously outlined, both theory of mind and learning and cognitive theories could help to explain this relationship (Shamay-Tsoory and Aharon-Peretz, 2007; Rousset et al., 2008). But it is also notable that we found a gender difference in results, with results showing a stronger relationship for females than for males. Previous studies have shown that females on average are more likely to read for pleasure and to consume fruit than males (Cooke and Wardle, 2005; Nippold et al., 2005). Future studies are needed to identify whether the influence of the choice of reading material amongst girls on their receptivity to positive narrative role models might explain results. For physical activity, an inverse relationship between reading and physical activity was not present cross-sectionally but was present longitudinally. This finding echoes a previous study on the negative relationship between sedentary behaviour (including reading books/magazines) and the levels of physical activity (Salmon et al., 2003). So the observed relationship may be explained by children's personal preference for reading displacing time available for other activities. In our fully-adjusted models, neither the relationship with cigarette use nor alcohol consumption were maintained when accounting for all identified factors. However, it is notable that our models included some factors that could be considered to lie on the causal pathway rather than merely explaining baseline associations. When only adjusting for factors that were unlikely to lie on the causal pathway, we found that the association was still present for cigarette use. Therefore, future studies are required to test whether some of the factors explored here may in fact act as causal mediators to explain the longitudinal associations presented.

A major strength of the study is that the results were based on a nationally representative sample of the British population, with a follow-up interview when the sample reached young adolescence; a critical period when individuals begin to develop health-related habits and lifestyle that could affect trajectories of later health (Dawson et al., 2008; DeWit et al., 2000; Lien et al., 2001). Our study also benefited from the rich data collection that includes a broad range of information on respondents' circumstances surrounding their reading habit and lifestyle, allowing us to identify potential underlying mechanisms by performing nested models. However, as this study is observational, causality cannot be assumed. We controlled for a large set of control variables. But other factors could still explain the association we found. While we controlled for multiple aspects of children's mental health, development, adjustment, behaviours, wellbeing, and self-esteem, we were unable to control specifically for personality, so it remains possible that children who read more frequently have less neurotic personality types and are therefore less likely to engage in risk-taking behaviours. Additionally, we controlled for a broad range of factors relating to parent-child relationships, parent-child engagement, parental encouragement of reading and parental behaviours. However, we were unable to identify whether parents discussed health behaviours with their children. So it is possible that children who read more lived in households where parents had more open discussions about health issues. Nevertheless, these results suggest that there is an association between reading for pleasure and healthy behaviours that is worthy of further study to assess whether it could be causal.

Additionally, although we focused on ‘reading for pleasure’, the type and content of books (e.g. picture books vs. novels or fact vs fiction) was not considered in the study as this information was not captured in the data set. It is likely that a change in children's behaviour depends on the content of the materials they read; for instance it has been suggested that fiction books may have a greater influence on readers' emotions, behaviour and social abilities than non-fiction literature (Johnson, 2012; Mar and Macrae, 2006). This may result in heterogeneity in findings between studies that explore the effect of reading on health-related behaviours. Consequently, future studies are needed to explore the genre of books read by children. Nevertheless, our study sheds new lights into potential additional benefits reading for pleasure may have, which goes beyond from the conventional focus on academic performance in previous research (OECD, 2002; Johnson, 2012). Second, the measurement of fruit consumption and exercise was adequate but did not provide a level of detail that allowed more sophisticated sensitivity analyses. Further, the respondent on children's fruit consumption changed between Sweep 5 and Sweep 6 (parents in Sweep 5 and adolescents in Sweep 6), so there may have been response bias and the honesty of reporting is undeterminable using this dataset. In addition, young people's vegetable consumption was not asked in Sweep 5, so we only considered fruit consumption in our study. Future studies that are interested in the association between young people's reading and health-related behaviours may wish to use different data sets and examine the relationship between reading for pleasure and broader dietary variables such as unhealthy eating. It is also unknown whether children had access to either cigarettes or alcohol and what impact access had on their likelihood of engaging. Finally, we were limited to analyses at age 11 and 14 by the waves of available data from MCS. Future studies could consider more closely the optimal sensitivity periods for reading and health behaviours in children and adolescents.

## 5 Conclusions

In conclusion, this paper adds to evidence on the literature on the potential health benefits of reading by showing there is a longitudinal association with health-related behaviours, including a lower odds of early onset of cigarette and alcohol use and a higher odds of having two portions of fruit per day. It is timely given that interventions to encourage children to read for pleasure are being actively encouraged for broad wellbeing benefits (DfE, 2015). The data from this study suggest there could be a value to conducting intervention studies promoting reading amongst those with low frequency of reading to explore if there is a potential causal relationship with health-related behaviours.

## Declaration of competing interest

Both authors declare no conflicts of interest.

## Appendix A Supplementary data

Supplementary material Image 1
