# Supplementary material for: Reading for pleasure in childhood and adolescent healthy behaviours: Longitudinal associations using the Millennium Cohort Study
Source: Prev Med. 2020 Jan;130:105889. doi: 10.1016/j.ypmed.2019.105889 (PMC6983940; doi:10.1016/j.ypmed.2019.105889)
Supplement: Supplementary file 1 — Supplementary material [file mmc1.docx]

**Supplementary Figure 1 A flow chart of the analytical sample**

Total N in Sweep 5:

N = 13,469

Exclude participants who did not answer all outcome variables at Sweep 6 were included (13.3%)

Excluded participants who did not live with natural parents (3%)

Exclusion of participants who were born as twins or triplets (<1.5%)

Final inclusion:

N = 11,180

N = 12,889

N = 13,287

| **Supplementary Table 1 The association between reading at age 11 and health behaviours at age 14: an interaction term of reading and children’s gender (N=11,108)** | | | | |
| --- | --- | --- | --- | --- |
| **Explanatory factors** | **Odds ratio** | **95% CI** |  | **Percentage of association explained** |
| **Ever used cigarettes** | | | |  |
| Basic model (reading, gender, ethnicity) | 0.93 | 0.86-1.01 |  |  |
| = all | 0.95 | 0.87-1.03 |  | 28.6% |
| **Ever used alcohol** | |  |  |  |
| Basic model (reading, gender, ethnicity) | 0.96 | 0.87-1.06 |  |  |
| = all | 0.97 | 0.87-1.08 |  | 25.0% |
| **Two portions or more of fruit consumption per day** | | | |  |
| Basic model (reading, gender, ethnicity, baseline ) | 1.13 | 1.02-1.25 |  |  |
| = all | 1.12 | 1.01-1.23 |  | 7.69% |
| **Five or more days of moderate to vigorous physical activity per week** | | | | |
| Basic model (reading, gender, ethnicity, baseline) | 1.04 | 0.95-1.13 |  |  |
| = all | 0.94 | 0.89-0.99 |  | -250% |
| Note: Basic model controlled for gender, ethnicity (and children’s baseline fruit consumption/physical activity in the models estimating later fruit consumption and physical activity). The Demographic Factors model additionally adjusted for parents’ education, household income, parents’ employment status, and parents’ marital status. The Child Development model additionally controlled for children’s behaviours (prosocial behaviour, emotional problems, peer problems, conduct problems, and hyperactivity/inattention) and children’s educational performance. The Child Mental Health model additionally controlled for children’s depressive symptoms, subjective well-being and self-esteem. The Family Relationships model additionally controlled for the closeness of parent-child relationship, frequency of arguments between parents and children and frequency of playing active games with parents. The Peer Influence model additionally adjusted for the number of books at home, frequency of parents’ reading for pleasure, frequency of children going to the library and frequency of visiting a social networking website (and parents’ and peers’ cigarette use and alcohol use in the smoking and drinking models). | | | | |

| **Supplementary Table 2a The association between reading at age 11 and two portions or more fruit consumption per day at age 14: by gender (N=11,108)** | | | | |
| --- | --- | --- | --- | --- |
| **Explanatory factors** | **Odds ratio** | **95% CI** |  | **Percentage of association explained** |
| ***Female (N=5,609)*** | | | |  |
| Basic model (reading, ethnicity) | 1.31 | 1.21-1.42 |  |  |
| = all | 1.15 | 1.06-1.25 |  | 51.6% |
| ***Male (N=5,499)*** | |  |  |  |
| Basic model (reading, ethnicity) | 1.16 | 1.09-1.23 |  |  |
| = all | 1.07 | 1.00-1.14 |  | 56.3% |
| Note: Basic model controlled for gender, ethnicity and baseline fruit consumption. The Demographic Factors model additionally adjusted for parents’ education, household income, parents’ employment status, and parents’ marital status. The Child Development model additionally controlled for children’s behaviours (prosocial behaviour, emotional problems, peer problems, conduct problems, and hyperactivity/inattention) and children’s educational performance. The Child Mental Health model additionally controlled for children’s depressive symptoms, subjective well-being and self-esteem. The Family Relationships model additionally controlled for the closeness of parent-child relationship, frequency of arguments between parents and children and frequency of playing active games with parents. The Peer Influence model additionally adjusted for the number of books at home, frequency of parents’ reading for pleasure, frequency of children going to the library and frequency of visiting a social networking website. | | | | |

| **Supplementary Table 2b The association between reading at age 11 and five or more days moderate to vigorous physical activity per week at age 14: by gender (N=11,108)** | | | | |
| --- | --- | --- | --- | --- |
| **Explanatory factors** | **Odds ratio** | **95% CI** |  | **Percentage of association explained** |
| ***Female (N=5,609)*** | | | |  |
| Basic model (reading, ethnicity) | 1.00 | 0.94-1.08 |  |  |
| = all | 0.98 | 0.91-1.05 |  | 0.00% |
| ***Male (N=5,499)*** | |  |  |  |
| Basic model (reading, ethnicity) | 0.96 | 0.91-1.01 |  |  |
| = all | 0.93 | 0.88-0.99 |  | -75.0% |
| Note: Basic model controlled for gender, ethnicity and baseline physical activity. The Demographic Factors model additionally adjusted for parents’ education, household income, parents’ employment status, and parents’ marital status. The Child Development model additionally controlled for children’s behaviours (prosocial behaviour, emotional problems, peer problems, conduct problems, and hyperactivity/inattention) and children’s educational performance. The Child Mental Health model additionally controlled for children’s depressive symptoms, subjective well-being and self-esteem. The Family Relationships model additionally controlled for the closeness of parent-child relationship, frequency of arguments between parents and children and frequency of playing active games with parents. The Peer Influence model additionally adjusted for the number of books at home, frequency of parents’ reading for pleasure, frequency of children going to the library and frequency of visiting a social networking website. | | | | |

| **Supplementary Table 3 The association between reading at age 11 and health behaviours at age 14: only adjusting for factors that could not lie on the causal pathway (N=11,108)** | | | |
| --- | --- | --- | --- |
| **Explanatory factors** | **Odds ratio** | **95% CI** |  |
| **Ever used cigarettes** | 0.92 | 0.87-0.97 |  |
| **Ever used alcohol** | 0.96 | 0.92-1.00 |  |
| **Two portions or more of fruit consumption per day** | 1.15 | 1.09-1.20 |  |
| **Five or more days of moderate to vigorous physical activity per week** | 0.95 | 0.91-0.99 |  |
| Note: Controlled for gender, ethnicity, children’s baseline fruit consumption/physical activity (in the models estimating later fruit consumption and physical activity), parents’ education, household income, parents’ employment status, parents’ marital status, closeness of parent-child relationship, frequency of arguments between parents and children, frequency of playing active games with parents, frequency of parents’ reading for pleasure and parents’ and peers’ cigarette use and alcohol use (in the smoking and drinking models). | | | |
